# Food-borne Botulism Caused by Clay Cheese: A Case Report

**DOI:** 10.22114/ajem.v0i0.145

**Published:** 2019-06-18

**Authors:** Zhila Farzinpoor

**Affiliations:** Department of Infectious Diseases, Faculty of Medicine, Kurdistan University of Medical Sciences, Sanandaj, Iran.

**Keywords:** Botulism, Cheese, Clay, Food Packaging

## Abstract

**Introduction:**

Recently, the use of metal and plastic containers instead of clay containers in producing this type of cheese has provided the anaerobic condition for growing the bacterium and producing the botulinum toxin. In this case report was to introduce “clay cheese dug in the ground” as a source of botulinum toxin for the first time.

**Case Report:**

A 34-year-old man with dizziness, asthma, and inability to swallow for four days referred to the hospital emergency department. He had diplopia and ptosis for two days. During admission to the emergency, the patient was conscious without fever, but with dysarthria and bilateral ptosis, an impaired gag reflex, slow right papillary reaction to light, a decreased eye movement, and a decreased power of facial muscles and limbs. The patient reported the use of clay cheese in a week before referring to the emergency. Hematological, biochemical, electrocardiogram, magnetic resonance imaging, and chest X-ray assays were normal. According to the Centers for Disease Control and Prevention classification, these symptoms were related to botulism. The evaluation of serum samples, emission, and gastric juice confirmed botulism by type A toxin.

**Conclusion:**

Considering the clinical results of this case study, clay cheese, which is produced in the west of Iran, can be introduced as a new source of the botulinum toxin.

## Introduction

Botulism is a disease caused by a neurotoxin called Botulinum Toxin (BT) produced by the anaerobic bacterium named *Clostridium botulinum*. Although it has a low prevalence, it is a dangerous disease with debilitating outcomes. When BT enters the body, it controls the release of Acetylcholine and avoids the neurotransmitter availability to muscles, leading to the muscle paralysis (1). The Center for Disease Control and Prevention (CDC) has established a supervision system called The National Botulism Surveillance System in America since 1970. This center aims to gather records and follow all cases affected by BT. The center divides the BT into four types based on the way of entering the body. They consist of A) infant botulism in which the bacterium is spread in the digestive system and produces BT; B) food-borne botulism in which the bacterium is spread and grown in food and produces BT; C) wound botulism in which the wound is infected by the bacterium, followed by bacterium spread and BT distributes in the body; and D) other botulism types with unknown cause of poisoning, such as overdose while using BT for medical purposes. Adult intestinal toxemia as a rare case happens when BT enters the body in a way similar to infant botulism (2, 3). The CDC has reported 39 (20%) cases of food-borne botulism with 4% of death during 2015 (3). As the most common source of botulism is the use of canned food (because of its acidic property) (4), the aim of this case report was to introduce “clay cheese dug in the ground” as a source of botulinum toxin for the first time.

## Case presentation

A 34-year-old man with dizziness, dyspnea, and inability to swallow referred to the hospital emergency department. The patient presented with dizziness, followed by dyspnea, a gradual feeling of dryness in the throat, an impaired reflex, slow papillary reaction to light, a decreased eye movement, a decreased power of facial muscles and limbs, normal deep tendon reflexes (DTR), lack of focal neurologic signs, and respiratory failure. The patient reported the use of clay cheese in the week before referring to the emergency. In the laboratory evaluation, hematological and biochemical tests such as complete blood cell counts, and also serum level of sodium, potassium, blood sugar, BUN, and creatinine were normal. electrocardiogram, magnetic resonance imaging, and chest X-ray were also reported as normal. The patient was under supportive therapy, respiratory care, cardiac monitoring, and pulse oximetry after transferring to the intensive care unit (ICU).

Based on the clinical suspicion to botulism, diagnostic evaluations, including the evaluation of serum samples, emission, and gastric juice, were done to confirm the BT. The patient’s history showed the use of cabbage salad, vegetables, and clay cheese in the last week. Thus samples of these foods were evaluated regarding the BT. The patient was treated with three doses of antitoxin. Neurology consultation was done a day after hospitalization. After three days, the patient was transferred to the infectious diseases ward. After 11 days and resolving of ptosis, the power of organs reached the normal state (5/5), and the gag reflex was decreased; consequently, he was discharged with medication orders. Four days later, the BT test in blood and cheese samples came positive (toxin B) while it was negative in other food samples.

The patients’ wife also referred to the hospital with asthma and a feeling of dryness in the throat, followed by the inability to swallow, feeling of vomiting, and trouble speaking for four days. During admission to the emergency, the patient was conscious without a fever but, with dysarthria and bilateral ptosis, an impaired gag reflex, slow right papillary reaction to light, a decreased eye movement, a decreased power of facial muscles and limbs, normal DTR, and lack of focal neurologic signs and respiratory failure. This patient underwent supportive therapy and antitoxin prescription. In addition to these two patients, seven other members of the family referred to the hospital with the same signs of botulism to be treated.

## Discussions

Clostridium botulinum is an anaerobic microorganism and spore-forming bacillus that is found in the soil. It includes three types A, B, and C. This bacterium produces seven types of toxin A, B, C, D, E, F, and G with similar pharmacological but different serological properties. Human botulinum is caused by type C that produces four toxins A, B, E, and F (5–8). Clay cheese is introduced in this report as a source of BT for the first time. Clay cheese is a kind of traditional cheese produced in the Northwest of Iran. In the past, fresh cheese was collected in clay dishes and kept closed for six months under the ground. Nowadays, this type of cheese is produced in plastic or metal containers that provide the anaerobic condition for growing the bacterium and producing the toxin (7).

Clinical signs of food-borne botulism include a range of neurologic signs of insensible weakness in motor or cranial nerves to severe respiratory failure or respiratory arrest; all of them are caused by the blockade of the neuromuscular junction in autonomic nerves (8, 9). Clinical signs are the same in all types of BT, but the severity and lethality are more in type A than in others (9). Thus this type is more important clinically. In the present case report, the recognized toxin was type A. Although food-borne botulism does not necessarily have digestive signs, constipation is the most common symptom. Furthermore, abdominal cramps, diarrhea, and vomiting may also be observed. The first neurologic signs are dry mouth, blurred vision, and double vision, followed by dysarthria, dysphonia, and dysphagia. The first digestive and neurologic signs occur within six hours to eight days (10). In the present case study, the above-mentioned sings happened at the same duration. Digestive and neurologic signs included dysarthria and bilateral ptosis, an impaired gag reflex, slow right papillary reaction to light, a decreased eye movement, and a decreased power of facial muscles and limbs. The clinical recovery takes from several weeks to several months because of a synopsis recovery in neuromuscular junction following supportive treatment such as mechanical ventilation (11, 12). In this case report, the patient was discharged 11 days after the supportive treatment and recovered from clinical signs, which is consistent with the above-mentioned remarks.

Nowadays, metal and plastic containers are used to produce clay cheese that provides an anaerobic condition for toxin production. Thus producing clay cheese in this way is dangerous, which necessitates the required training for people involved with the production of this kind of cheese.

## Conclusions

Considering the clinical results of this case study, clay cheese, which is produced in the west of Iran, can be introduced as a new source of the botulinum toxin. This phenomenon can be due to changing the method of producing this type of cheese.
